# Pharmacogenomics in Children: Advantages and Challenges of Next Generation Sequencing Applications

**DOI:** 10.1155/2013/136524

**Published:** 2013-01-17

**Authors:** O. M. Vanakker, A. De Paepe

**Affiliations:** Center for Medical Genetics, Ghent University Hospital, De Pintelaan 185, 9000 Ghent, Belgium

## Abstract

Pharmacogenetics is considered as a prime example of how personalized medicine nowadays can be put into practice. However, genotyping to guide pharmacological treatment is relatively uncommon in the routine clinical practice. Several reasons can be found why the application of pharmacogenetics is less than initially anticipated, which include the contradictory results obtained for certain variants and the lack of guidelines for clinical implementation. However, more reproducible results are being generated, and efforts have been made to establish working groups focussing on evidence-based clinical guidelines. For another pharmacogenetic hurdle, the speed by which a pharmacogenetic profile for a certain drug can be obtained in an individual patient, there has been a revolution in molecular genetics through the introduction of next generation sequencing (NGS), making it possible to sequence a large number of genes up to the complete genome in a single reaction. Besides the enthusiasm due to the tremendous increase of our sequencing capacities, several considerations need to be made regarding quality and interpretation of the sequence data as well as ethical aspects of this technology. This paper will focus on the different NGS applications that may be useful for pharmacogenomics in children and the challenges that they bring on.

## 1. Introduction

Pharmacogenetics refers to the influence of DNA variants on drug response, the knowledge of which can facilitate selection of the optimal drug, dose, and treatment duration and avert adverse drug reactions [1]. Several demonstrations have been given on the differences in response to drugs between children and adults [2]. These include differences in drug metabolism and gene expression, the latter being a highly dynamic process functioning from the neonatal period over childhood into adult life. Though the number of studies specifically devoted to the pediatric population is still limited compared to adults, an increasing number of genes are being identified in which variants have an influence on pharmacological treatment of childhood diseases [3]. The identification of variants in novel genes as well as the validation of their functional effects will further increase our ability to predict drug treatment response in children; at the same time, the clinical implementation of this knowledge will demand an efficient diagnostic approach to first identify a pharmacogenomic profile in an individual patient in a short period of time, next to evidence-based clinical guidelines to facilitate decision making based on the genotype [4].

The current golden standard for detecting pathogenic variants—single nucleotide variations or small indels—is Sanger sequencing [5, 6]. Developed in the late 70s by Frederick Sanger, an English biochemist, the technique has currently been optimized to evaluate variations in PCR-amplified DNA fragments with high sensitivity and specificity. The major disadvantages of Sanger sequencing—particularly in a domain such as pharmacogenetics where for a specific drug variants in multiple genes can be, either independent of or in interaction with each other, involved—are that each novel genetic test needs optimization and turn-around times for each gene analysis can be relatively long, certainly if therapeutic decisions would be based on these results. Together with the sometimes ambiguous evidence for the effect of certain variants and the lack of robust validation and clinical guidelines, this technical hurdle has been one of the reasons that genotyping to inform clinical decisions regarding pharmacological treatment is not widely practiced to date.

The introduction of next generation sequencing (NGS) brought about a technological revolution among genetic screening tools, as it now becomes possible to screen the whole exome—the coding regions of our DNA—and even the complete genome in a single experiment [7–9]. The increase of technological capacities and decrease of costs involved in such analysis have resulted in successful implementation of exome sequencing as a research tool, particularly to identify novel genes for rare disorders [10, 11]. Causal genes for, for example, the Freeman-Sheldon (OMIM no. 193700) or the Kabuki syndrome (OMIM no. 147920) were identified by combining whole exome sequencing data from different patients with a typical phenotype of these conditions [12]. They demonstrate that it is possible to capture exomic variation and identify pathogenic variants using bioinformatic tools. Since then, several other examples have been reported.

Because of this success, these screening techniques are slowly starting to make their way as a diagnostic tool. Certainly for complex diseases for which several genes have been identified—the sequence analysis of which is laborious, time consuming, and expensive—the idea of sequencing all 23.000 genes in the exome in a single reaction is an alluring alternative. Similarly, in a field such as pharmacogenetics, with different variants in different genes influencing the final drug response in an individual patient, such parallel sequencing techniques can provide the promptness which would be required in a clinical setting. This shift to the more extensive screening assays has induced an evolution from pharmacogenetics to pharmacogenomics [3]. Besides the excitement surrounding these technical innovations, it has become clear that NGS applications also present several challenges. These include not only the quantity of data which is generated, its analysis, and interpretation but also ethical and legal aspects. In the pediatric population, the latter have very particular properties as a consequence of the incapacity of the child to give informed consent himself and of the predictive character of the interpreted sequence data which may go beyond the initial clinical question.

In this paper, we will consider the characteristics of NGS, the different means by which NGS technology can be applied, and set out a concept that we think would be feasible to use NGS-based pharmacogenetics in a present-day clinical pediatric setting.

## 2. Genomes, Exomes, and the Variation within

The human genome is the entirety of an individual's hereditary information, including both the coding and noncoding regions of DNA and RNA, while the human exome encompasses the coding regions of the genes—the exons—equivalenting ~1%-2% of the total haploid genomic sequence [12, 13]. Since the establishment of the reference genome and subsequent sequencing of several individual genomes, insights have emerged on the significant variation present in the genome within and between different ethnicities. This variation can be roughly divided into simple nucleotide variations on one hand and structural variation on the other [12]. The first include single nucleotide polymorphisms (SNPs) and small insertions/deletions (indels) which have been surveyed in large groups of individuals, resulting in, for example, dbSNP, a database of over 10 million common variants in different ethnic groups. Increased knowledge on the architecture of the genome revealed, however; the spectrum of variation was much broader than these nucleotide changes, referred to as structural variation. This includes not only inversions and copy number variations (CNVs, i.e., deletions and duplications) but also, for example, the presence of stretches of megabases of DNA unique to a single personal genome. It has become clear that structural variation in the genome is unexpectedly high and much more complex than previously anticipated (Table 1). In pharmacogenomics, both single nucleotide variants and structural variation such as CNVs have been shown to contribute to the drug response of an individual [14, 15]. The pleiotropy by which this variation occurs has an impact on the identification of functional variants for drug response and on the analysis and interpretation of genomic screening assays.

## 3. Next Generation Sequencing

**Figure 1:**
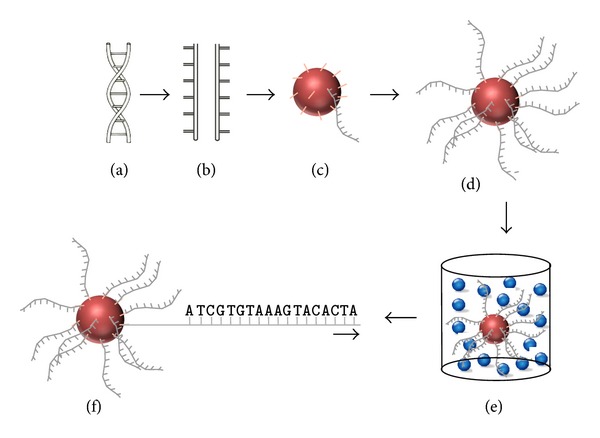
Schematic representation of the principles of massive parallel sequencing using beads. Double-stranded DNA (a) is fragmented into single-stranded DNA (b) which is subsequently coupled, via adaptors, to agarose beads by oligonucleotides complementary to the adaptor (c). These beads are submerged in an emulsion where amplification of the single DNA fragment occurs (d). Subsequently, the bead is placed into a well (e), already equipped with all reagents for sequencing (small beads). Within these wells, parallel sequencing of the different DNA fragments occurs, resulting in the generation of the genetic code of the fragment (f).

Following the Human Genome Project, which set out to sequence the three billion nucleotides of the human genome, several high throughput technologies were developed. Among these, NGS has known a rapid evolution in a few years time, increasing throughput and reducing costs by continuous improvement of several analysis platforms [7, 9, 16, 17]. Although all are based on the principle of massive parallel sequencing, the specific workflow of NGS depends on the platform that is being used; one of these techniques which demonstrates excellently the meaning of massive parallel sequencing was developed by Margulies et al. (Figure 1) [6, 9, 18]. In summary, after fragmentation of the genomic DNA, these fragments are bound to tiny beads under specific conditions so that only one DNA fragment can bind to a specific bead. These beads are encased in droplets of oil, containing all reactants necessary to amplify the DNA via polymerase chain reaction (PCR). In this way, each bead ends up with about 10 million copies of the initial DNA fragment. For sequencing of these fragments, the beads are loaded into a well plate—one bead per well—and sequenced by “the sequencing-by-synthesis method” (determination of the sequence by addition of nucleotides to the complementary strand). Again, all reagents for the sequencing reaction are already present in each well.

General limitations of massive parallel sequencing include error rate, which is higher than Sanger sequencing, warranting confirmation of identified causal variants by conventional sequencing methods [6, 11, 19]. Further, the quality of the sequence data or so-called coverage (i.e., to which extent is every nucleotide of the sequence of interest—whether it is a selected set of genes or the whole genome—reliably analyzed) depends on the sequence depth. Valid results can be obtained from 40- to 80-fold sequence depth, meaning that every nucleotide is sequenced 40 to 80 times [9, 20]. The higher the coverage, the more reliable the result, but also the more expensive and laborious will the analysis be as more sequencing needs to be done. The importance of optimizing coverage of current NGS assays in pharmacogenetics was recently demonstrated in a meta-analysis which evaluated the efficiency of current platforms in the analysis of 253 pharmacogenes. It was shown that a maximum of 85% of coverage of these genes could be obtained, while maximally 30% of missense polymorphisms were covered [20]. This underscores the limitations of genome-wide methods and the challenges and priorities for further optimizing NGS assays.

## 4. Applications of NGS

NGS can be used in a targeted manner or can be applied as a whole exome of whole genome diagnostic tool. Every one of these approaches has its advantages and weaknesses which will be discussed in the following, with respect to pharmacogenomics in children.

### 4.1. Targeted Assays

In a targeted assay, NGS is used for parallel sequencing of a selection of genes. There are two ways to go about selecting the genes of interest, which determines the molecular technique that will be applied: either a microarray-based target enrichment approach or a targeted analysis of whole exome/genome sequencing (WES and WGS, resp.) can be used. In the first, direct hybridization of the patient's DNA to an oligonucleotide array, containing probes complementary to the selection of target genes, is performed and then analyzed by NGS, thus generating sequence data only of the genes of interest [16, 21]. In the second approach, sequence data of the complete exome or genome is generated, but afterwards only the specific genes of interest are bioinformatically selected and analyzed further, while the remaining sequence is disregarded [9, 17].

**Figure 2:**
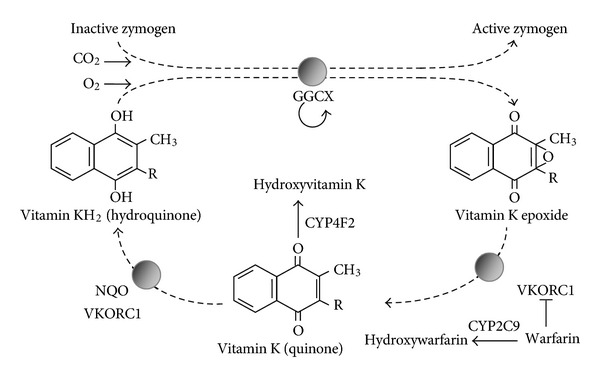
Vitamin K cycle and warfarin metabolism. Inactive zymogens, among which are the VK-dependent coagulation factors II, VII, IX, and X, are activated by gamma-carboxylation by the gamma-glutamyl carboxylase (GGCX). The cofactor for this carboxylation step is VK, which is transformed into VK epoxide. This epoxide is then reduced by the VK-epoxide reductase of VKORC1 to quinone. Warfarin specifically blocks the initial reduction step, while CYP4F2 catalyzes the formation of hydroxyvitamin K out of quinone.

The major advantage of the array-based target enrichment assays is that these selective tests can be optimized to have full coverage of the genes of interest, hence reaching high sensitivity and specificity. Moreover, for each gene it is possible to design the array to just cover the sequence (exon, intron or promotor) in which a particular SNP is present. Further, by generating sequence data only of the genes of your interest, the risk for incidental findings or ethical issues on generated but unanalyzed sequence data—both discussed in the following—is minimized. A good pharmacogenomic example of such an analysis would be coumarine treatment. The drug response to warfarin—a paradigm for drugs with a narrow therapeutic index—is determined by variants in several genes, including VKORC1, GGCX, CYP2C9 and, CYP4F2 [22–24]. Among these, VKORC1 and GGCX encode key enzymes (VK-oxide reductase and gamma-glutamyl carboxylase; resp.) of the vitamin K (VK) cycle, the metabolic process—essential for activation of VK-dependent coagulation factors II, VII, IX, and X—which is blocked by warfarin (Figure 2) [25]. Cytochromes P450 2C9 and P450 4F2 are important in the metabolization of the drug, catalyzing the warfarin S-enantiomer into its inactive metabolites (CYP2C9) or oxidase VK1, the essential cofactor of the VK cycle (CYP4F2) [24, 26].

An important observation is that the functional variants in, for example, the VKORC1 gene reside mostly in noncoding regions such as the introns and promotor of the gene [22]. Contrary to exome analysis, where only the coding regions are sequenced, a targeted array-based assay can be maximally optimized to cover both the coding and non-coding regions of these 4 genes, thus resulting in a maximum of relevant information in a single reaction.

Nevertheless, it must be remembered that drugs such as warfarin, where most of the variance in metabolism and clearance can be captured by analyzing a handful of genomic variants, represent only a proportion of drugs with a narrow therapeutic index; for many other drugs, this will not be the case, confronting us with the main limitation of this type of targeted analysis which is the limited flexibility in design. It can be expected that for many drugs, novel genes and variants will be discovered which will have a pharmacogenomic effect in addition to the ones known to date. Though this number may be rather small for a very specific topic such as warfarin biology, the pharmacogenetics of ADHD or asthma treatment—for both of which variants in non-coding regions were described (Table 2)—will likely expand significantly in the years to come [3, 27–30].

This implies that with every newly identified gene, the assay needs to be adjusted or updated to obtain the highest yield of useful information. Though novel generation arrays have already become more “user friendly” to expand the number of targeted genes, it still does not come near the ease by which additional genes can be analyzed in a prospective way using WES or WGS. When applying targeted exome analysis, whole exome sequencing is performed resulting in sequencing data of 23.000 genes. Subsequently, only those genes of interest are filtered out to analyse variants. When a novel gene is identified, it is easy to go back to the initial sequence data, access the sequence of the new gene, and analyze variants. However, as mentioned, the major limitation of this approach is the lack of good sequence data of non-coding regions, making this technique only useful to obtain a pharmacogenetic profile for those drugs of which the relevant variants are in coding regions. One such example is the treatment of acute lymphoblastic leukemia (ALL) in children [2, 31]. As chemotherapeutic agents are often given at a dose near the toxic range and significant interindividual variability can be seen in effect and adverse reactions, pharmacogenetics can aid in tailoring treatment to the specific needs of the patient. Several polymorphisms in enzymes which metabolize chemotherapeutics have been shown to alter treatment response (Table 3), all of which affect the coding regions of the respective genes. In ALL patients, targeted WES can be an option as all relevant variants will be covered.

### 4.2. Whole Genome and Exome Analysis

Since the completion of the Human Genome Project in 2001, sequencing of the complete personal genome has become a technical reality [12, 13]. Since the initial assembly, the reference genome has been refined and provided us initial insights in to the complexity and extent of human genetic variation. Since then, initiatives such as the 1000 Genomes Project aim to further characterize human variation of all types in different ethnic populations [8]. Similar to exome sequencing, the true challenge of whole genome analysis lies in the identification of disease causing mutations or functional SNPs among an average of 3.0–3.5 million SNVs and *∼*1000 CNVs in a human diploid genome [12].

### 4.3. (Targeted) Whole Genome Sequencing

The current feasibility of pharmacogenetic implementation of WGS data has been shown in published personal genomes such as the Lupski or the Venter genome [7, 12]. In both, several variants were identified with clinical pharmacogenetic significance, including warfarin or clopidogrel sensitivity. Another recent example on how WGS can aid therapeutic treatment came from a pair of twins suffering from dopa-responsive dystonia. Their genomes were analyzed, and—assuming recessive inheritance—the list of candidate genes was narrowed down to three. One of these was sepiapterin (SPR), a gene previously reported in association with DRD (OMIM no. 612716) but so rare that no specific diagnostic test exists. Importantly, patients with SPR mutations also have insufficient tetrahydrobiopterin (BH_4_), an important cofactor in the biosynthesis of dopamine and serotonin. Treatment of these patients with L-dopa and 5-hydroxy-tryptophan resulted in marked clinical improvement [32]. Though in this particular case unrestricted WGS was applied, it can be conceived that targeted WGS focussing on all genes known to be related to DRD could be used to improve diagnostics and choose the optimal treatment strategy.

### 4.4. Whole Genome Analysis as the Standard of Clinical Care

One of the issues that has risen recently is whether the characterization of the genomic sequence of an individual should become the standard of care. This issue has great relevance to the pediatric population, and several arguments can be conceived why this data should or should not be available as early on as possible, ideally in the neonatal period.

There can be two rationales to gain knowledge of this genomic information: the first would be to improve preventive medicine by identifying causal mutations or risk alleles associated with so-called actionable diseases, that is, diseases for which preventive measurements of screening have been shown useful for improvement of prognosis. After sequencing, the whole genome data set is completely analyzed, with the intrinsic risk to also unveil risk alleles or mutations for nonactionable disorders such as, for example, neurodegenerative diseases. The second rationale would be to have rapid access to the genetic background of an individual if there is an acute disease episode, for diagnostic and pharmacogenetic purposes. This would imply that the uninterpreted data is stored, and—when, for example, the patient develops symptoms of asthma—the sequences of those genes known to be involved in drug response of beta-agonists can be quickly assembled and searched for variants that may guide treatment options.

Though both scenarios can be seen as the ultimate refinement of personalized medicine, there are several practical, ethical, and legal considerations that need to be made before this can be implemented, most of which also apply for WES.

### 4.5. Practical Issues

**Figure 3:**
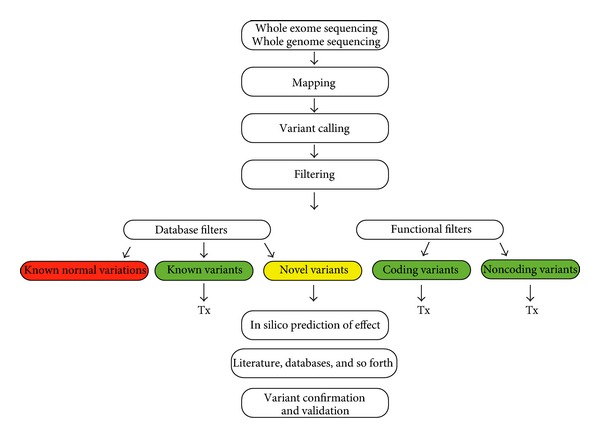
Workflow for pharmacogenomics using WES or WGS. After mapping to the reference sequence and variant calling, variants need to be filtered. Besides the known functional variants and the normal variations, it can be expected that a considerable number of novel variants in genes involved in drug metabolism will be found. For these, additional functional assays will have to be performed to confirm whether they have functional relevance or not.

Besides technical issues related to storage and access of the data and data analysis (method and quality of analysis as well as the bioinformatic—hard- and software—capacities), the main practical issue remains the interpretation of the sequence data (Figure 3) [7, 33–35]. The human genome is highly variable, with a difference of each personal genome from the reference assembly in 3.5 million SNPs and 1000 large CNVs. Moreover, it is considered that each personal genome contains 400.000 to 600.000 novel SNPs compared to databases such as dbSNP. Moreover, not only the functional effect of each individual variant should be considered but also the interaction between different variants. This has, for example, been shown for copy number variants, many of which can be found across genes encoding proteins with known drug-metabolizing activity. These deletions and duplications can have a high prevalence in the general population, ranging from 2% to 34%, and individuals who are considered outlier metabolizers (poor or ultra-metabolizers) were shown to harbor a high amount of these CNVs (deletions and duplications, resp.). However, the presence of such a CNV in a patient does not necessarily mean that it will be predictable for the metabolizer status of that patient. Besides the fact that many of these—particularly duplications—may be nonfunctional, their effect may be compensated by other variants or regulatory mechanisms, in the end leading to little change in the drug metabolism of that individual. Hence, decision-making for, for example, drug dosage based on this variant alone may result in under- or overdosing. This leads to the conclusion that extensive empirical evaluation of variants on drug metabolism in the general population will be needed before they can be applied in clinical routine and that postmarketing studies will also need to address this issue.

### 4.6. Ethical and Legal Issues

Several ethical considerations need to be made prior to routine clinical implementation, in adults but particularly also in children. The huge amount of personal medical data produced by NGS, the fact that some will be irrelevant, that some may be relevant for diseases beyond the primary reason for the test and that some may be difficult to interpret and hence unclear, make that all ethical issues raised before on genetic testing now come together in a single test [36].

A first issue is the consent. Because of its specific nature, certainly WES and WGS require a different kind of consent compared to the routine genetic tests. As mentioned previously, a potential benefit of these screening technologies for the patient may be the early detection of actionable diseases, the symptoms of which can occur in childhood. Besides the obvious advantages for followup and prognosis, it must be taken into account that being confronted unexpectedly with the knowledge that the child may develop one or more diseases can bring about significant psychological burden for the child and the parents. While this will be so for variants associated with high risk to develop disease, -suddenly, an additional medical track needs to be established for this novel health problem-, the psychological effect may be even more pronounced when a variant is discovered with mediocre or low penetrance and hence increased uncertainty about the future of the child. Therefore, it seems imperative that prior to WES or WGS for a given diagnostic question, the patient and/or his parents are informed about other diseases for which the test can reveal information and what the implications can be of each of these. The consent that is given will need to not only stipulate rigorously which diseases are being (indirectly) looked at, but also mention the possibility of variants of unknown significance, of which it remains uncertain what the effect may be. Needless to say that this will require a much more extensive pretest counseling compared to the molecular testing that is routinely used to date as well as posttest (psychological) followup.

To respect the right of the child not to know, it has been a standard policy not to perform presymptomatic tests in children, when the onset of the disease is in adulthood [34]. A broad screening assay such as untargeted WES or WGS in the context of pharmacogenomics for a childhood disease will also reveal information on these specific “late-onset” diseases so that one can reflect on whether parents who give consent for this analysis have the right to disregard the right not to know of their child. In contrast, the situation may occur that a mutation of a late-onset disorder was inherited from a parent who does not have any symptoms yet at the moment of the test. When it concerns an actionable disease, this knowledge may improve treatment and prognosis of this individual and may play an important role in the decision-making for future pregnancies. A similar situation may arise regarding the carriership of mutations for autosomal recessive disorders. Mutation analysis in obligate or potential carriers is in most cases not performed in childhood, as there are no implications for the health of the carrier, and the child has the right to decide himself/herself whether he or she wants to know their carrier status. Using untargeted WES or WGS, carriership of several autosomal recessive traits would be identified in every patient analyzed. One could argue to discard this information as it has no immediate benefit for the patient. On the other hand, this information may be important for the parents—if they would have another child—and possibly other family members, particularly if the carrier frequency in the general population is considerable. Should the right not to know of the child overrule the potential benefit for the parents and other family members? These situations can be seen as a plea for targeted analysis, as the risk for such incidental findings would be minimized. Though this poses few problems when the sequence data that is generated is limited to the genes of interest, but when targeted analysis is performed on a larger sequence data set—in the context of targeted WES or WGS—it should be considered that all sequence data on actionable diseases, although uninterpreted, is available. Does the child not have the right to know whether other genetic information in his genome is present for actionable diseases, the knowledge of which may influence his health in a significant manner? Or correspondingly, does the physician or the diagnostic lab not have the obligation to inform the patient? Though no conclusive answer can be given to these questions at this time, many uncertainties can be avoided if the informed consent form stipulates in detail not only which tests will be performed but also what will not be examined [37].

All issues mentioned previously underline that a thorough debate addressing the medical, ethical, and psychological aspects of WES and WGS with respect to the child, the parents and the rest of the family is necessary prior to the diagnostic implementation of these techniques in, for example, pharmacogenomics. Such debate has begun to occur within the genetic community, and international consensus and guidelines will need to be drafted regarding late-onset disorders, carriership of recessive diseases, and actionable or non-actionable childhood diseases. However, because of the extensive impact these screening strategies can have, a more global public debate is necessary to inform the public about these novel possibilities and their challenges and to think about what people really want to learn from such a genetic test when sufficiently informed.

A second issue focusses on the interpretation of sequence data. Should patients be informed of variants of uncertain significance? The main argument not to provide such details is that it does not give additional information to the patient at the time of consultation. However, as knowledge increases, more may become known about these variants; this could imply that what was once a variant of unknown significance may turn out to be of immediate relevance to the patients health or that of his family members. Knowledge of these variants, even when their meaning is initially unclear, may be useful in the followup. In this respect, the question whether the physician or diagnostic facility has the duty to recontact patients when the interpretation of their sequence data changes over the years has not been answered. If this were to be the case, a fully automated informatics system would be needed to regularly screen the stored genomic data of every patient and match all variants with the current literature. To our knowledge, such large-scale systems which are flawless are not yet available, making the duty to recontact for these large data sets nearly impossible at this time.

Legal issues that can arise around WES/WGS include the storage and access of genomic sequence data and the question of who can gain access: the individual himself, his treating physician(s), insurance companies, police, and so forth [35]. Also gene patenting might become an important subject.

## 5. Conclusion

The technical revolution in sequencing analysis tools has lead to new perspectives for personalized medicine in general and in pharmacogenetics/genomics specifically. Next generation sequencing and its applications have increased our ability to unravel the genetic code of an individual with significant improvement of the speed of the analysis. On the other hand, the implementation of these assays brings about several considerations regarding sensitivity, data analysis, and interpretation as well as ethical aspects. Of the current NGS technologies, the array-based approach seems to be the most feasible one for pharmacogenomic applications in childhood. Its targeted nature avoids incidental findings while offering sufficient coverage of coding and noncoding regions of genes of interest. With little doubt, the future perspective will be the application of WGS as a diagnostic tool, also in pharmacogenomics. However, many questions need to be addressed before implementing this screening technique in the clinic, including technical challenges, interpretation difficulties, and ethical considerations. Most importantly, the implementation of NGS requires the establishment of genotypes with clinical utility and guidelines on how to use them. Though the topic of research in many areas, more effort will have to go to validating genotypic data and developing clinical algorithms using them.

## Figures and Tables

**Table 1:** Genomic variation in an individual in numbers. All data is given for an individual exome or genome. The number of nonsynonymous variants, which induce a change in amino acid, has been specified as these are more likely to have a functional effect than synonymous variants (where the amino acid remains the same despite the nucleotide change), although functional synonymous variants have been described.

	Number of variants
Similarity between two individual genomes	99.5%
Whole genome sequencing variant uptake	3,5 million SNP variants 1000 large CNVs
Whole exome sequencing variant uptake	20.000–100.000 variants
Coding variants in the genome	20.000–25.000 variants
Nonsynonymous coding variants in the genome	9.000–11.000 variants

**Table 2:** Identified pharmacogenes in ADHD and asthma.

	Gene	Variants
Asthma	ADRB2	p.Arg16Gly, p.Gln27Glu
	AC9	p.Ile772Met
	CRHR1	c.122-1310C > A (intronic)
	TBX21	p.His33Gln
	LTC4S	g.24030224A > C (promotor)
	CYSLTR1	p.Phe309Phe
	ALOX5	5′ UTR
	GSDML	c.236-1199G > A (intronic)

ADHD	DRD4	48 bp allele
	DAT1	3′ UTR
	5-HTT	del-1212-1255 (promotor)
	SNAP-25	3′ UTR
	COMT	p.Val158Met
	ADRA2A	g.31585029G > C

ADRB2: agonists of beta-2 adrenergic receptor; AC9: adenylyl cyclase type 9; CRHR1: corticotropin releasing hormone receptor 1; TBX21: T-box 21; LTC4S: leukotriene C4 synthase; CYSLTR1: cysteinyl leukotriene receptor 1; ALOX5: arachidonate 5-lipoxygenase; GSDML: gasdermin B; DRD4: dopamine receptor 4; DAT1: dopamine transporter 1; 5-HTT: serotonin transporter; SNAP-25: synaptosomal-associated protein; COMT: catechol-O-methyltransferase; ADRA2A: adrenergic alpha2-receptor; UTR: untranslated region.

**Table 3:** Identified pharmacogenes in childhood ALL. All are affecting the coding regions of the respective genes.

Gene	Variants
TPMT	p.Ala80Pro, p.Ala54Tyr, p.Tyr240Cys
GSTT1	Large deletion
GSTM1	Large deletion
GSTP1	p.Ile105Val
MTHFR	p.Ala222Val, p.Glu429Ala
GGH	p.Thr151Ile

TMPT: thiopurine methyltransferase; GST: glutathione-S-transferase family; MTHFR: methylenetetrahydrofolate reductase; GGH: gamma-glutamyl hydrolase.
